# A new route for the syntheses of coordination polymers using magnetic influence: syntheses, crystal structures and fluorescence properties

**DOI:** 10.1107/S2052252523007650

**Published:** 2023-09-18

**Authors:** Lei Guan, Ying Wang, Hongzhe Jin, Pengpeng Yin

**Affiliations:** a Liaoning Petrochemical University, Fushun, Liaoning 113001, People’s Republic of China; King Abdullah University, Saudi Arabia

**Keywords:** coordination polymers, fluorescence, synthetic approach, transition metals, crystallization and crystal growth

## Abstract

Five coordination polymers [TM^1^(absa)(H_2_O)_4_]_*n*_ and [TM^2^(absa)(bipy)(H_2_O)]_*n*_ [TM^1^ = Zn (**1**), Co (**2**); TM^2^ = Zn (**3**), Co (**4**), Cu (**5**); Na_2_absa = 5,5′-azobissalicylic acid disodium salt; bipy = 4,4′-bipyride] were synthesized by solvent evaporation under a magnetic field. It is evident that magnetic fields bring significant and noticeable changes to the absa^2−^ ligand orientation and the component movement behaviors to construct coordination polymers. The absa^2−^ ligands bind to the metal ions in bridging coordination mode through the carboxyl­ate groups, in addition to the bipy molecules adopting bridging modes. Photoluminescence measurements indicate that the emissions of compounds **1**–**5** are at 626, 600, 632, 658 and 682 nm in the solid state, respectively.

## Abstract 2

Five coordination polymers were obtained based on an azo multifunctional aromatic ligand. The movement and collision behaviors of components are greatly affected by the magnetic field assisted method, which could have a subsequent impact on their structures with different space groups.

## 1. Introduction

The self-assembly of coordination compounds is induced by the coordination bonds between metal cations and ligands, as well as various weak intermolecular interactions, such as hydrogen bonding and π–π interactions *etc.* (Chen & Liu, 2015 ▸; Robson, 2000 ▸; Byrne *et al.*, 2008 ▸; Leong & Vittal, 2011 ▸; Huang, 2003 ▸). The self-assembly of these components in solution can construct varied coordination polymers and form diverse topological networks with high dimensionalities (Erxleben, 2003 ▸; Ghosh & Bharadwaj, 2004 ▸; He *et al.*, 2009 ▸). However, functional groups, coordination modes, charges and acidity have great effects on the final architectures, in addition to the ligands and metal cations (Kirillov, 2011 ▸; Zaworotko, 2010 ▸; Bitzer & Kleist, 2019 ▸). Therefore, the final structrual topologies induced by the weak interactions are not always unambiguously predictable and controllable. Recently, efforts have been focused on reliable synthetic strategies with the aim of obtaining predictable architectures with unusual topologies and physical properties (Lee *et al.*, 2017 ▸; Williams *et al.*, 2007 ▸). The magnetic field acts as a special driving force. The dynamical properties of the aqueous solution and colloidal system are changed under a magnetic field (Sheibani *et al.*, 2003 ▸). Additionally, the phase equilibrium relationships between components are broken (Hermann *et al.*, 2013 ▸; Gelfgat, 1999 ▸). Therefore, the use of magnetic influence has received considerable attention and has been applied in many fields, such as nanomaterials, biomedicine, environmental protection, metallurgy and semiconductors (Lin *et al.*, 2014 ▸; Serantes *et al.*, 2018 ▸; Cépas *et al.*, 2002 ▸). It has been found that a magnetic field has unexpected effects on the crystallization behavior of drug molecules, preparation of nano-materials, growth of bulk single crystals and the processes of chemical reactions (Hermann *et al.*, 2013 ▸; Ronco & Ferraudi, 1990 ▸). Consequently, our strategy – in order to gain control of and utilize the weak intermolecular interactions in solution – is to employ a controllable magnetic field as a driving force for constructing target products.

Herein, Na_2_absa was employed as the ligand in the design and construction of various architectures. It has three distinctive characteristics: (1) four functional groups, which can present a diverse number of potential coordination modes, allowing for the formation of diverse topologies; (2) a rigid and long molecular structure, which can give rise to the formation of interpenetrating frameworks; and (3) a π-conjugate system, which can provide a π-surface for intermolecular interactions and can be easily affected by a magnetic field. When the Na_2_absa ligand was used in combination with the rigid 4,4′-bi­pyridine (bipy) building block, five transition metal coordination polymers were synthesized via solvent evaporation under a magnetic field (see below). 
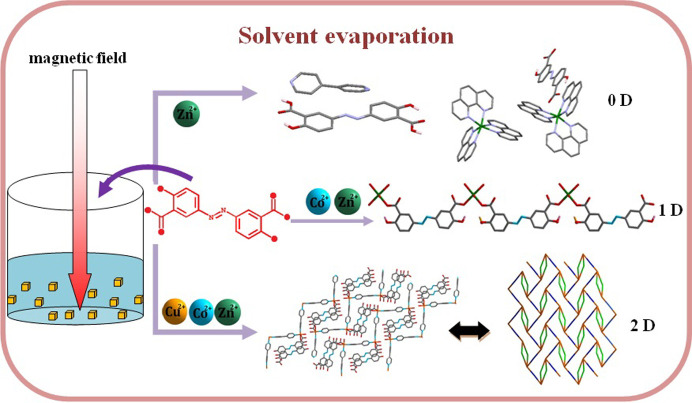


## 2. Experimental

### 2.1. Materials and general measurements

The reagents and solvents employed were commercially available and used as received without further purification. Single-crystal X-ray diffraction data were collected with a Rigaku Saturn 70 CCD, a Bruker APEX-II diffractometer or a Bruker D8 VENTURE TXS PHOTON 100 equipped with graphite monochromated Mo *K*α radiation (λ = 0.71073 Å) using either the ω or the φ–ω scan mode. Elemental analyses of carbon, hydrogen and nitro­gen were performed with a Perkin Elmer 240C elemental analyzer. The infrared spectra were measured by a Magna-IR 750 spectrophotometer in the 4000–400 cm^−1^ region (KBr pellet). Thermogravimetric analyses (TGA) were carried out on a NETZSCH STA 449C unit at a heating rate of 10°C min^−1^ under a nitro­gen atmosphere. Photoluminescence analyses were performed on a Perkin Elemer LS55 fluorescence spectrometer.

### 2.2. Syntheses of Na_2_absa ligand

5,5′-azobissalicylic acid (H_2_absa) was synthesized using procedures described in the literature (Kenawy *et al.*, 2010 ▸). Sodium hydroxide solution (10%) was added dropwise to H_2_absa (0.302 g, 1 mmol) with stirring to a pH value of 5, and the reaction mixture was further stirred for 1 h at room temperature. The product was recrystallized three times and colorless crystals were collected by filtration and dried under vacuum at room temperature.

### 2.3. Syntheses of compounds **1** and **2**

The corresponding metal nitrates (0.1 mmol) and Na_2_absa (0.1 mmol) were added to a vessel containing mixed solvent (5 ml of methanol and 10 ml of distilled water). The mixed solution was stirred and heated at 100°C for 3 h under a 1 T magnetic field. The solution obtained was kept in a small vial covered with parafilm at room temperature and left under a magnetic field. Block crystals were obtained after 10 days. For compound **1**, IR (KBr, cm^−1^): 3023, 1579, 1479, 1448, 1328, 1248, 1187, 1079, 828, 789, 720, 577, 473. Calculated for C_14_H_16_N_2_O_10_Zn (%): C, 38.39; H, 3.66; N, 6.40. Found (%): C, 38.42; H, 3.82; N, 6.46. For compound **2**, IR (KBr, cm^−1^): 3211, 1577, 1330, 1481, 1450, 1385, 1249, 1187, 1140, 1079, 853, 826, 792, 718, 677, 626, 575, 473. Calculated for C_14_H_16_N_2_O_10_Co (%): C, 38.96; H, 3.71; N, 6.49. Found (%): C, 38.90; H, 3.80; N, 6.55.

### 2.4. Syntheses of compounds **3**–**5**

Compounds **3**–**5** were synthesized via a similar procedure used to produce compound **1** with the corresponding metal nitrates (0.1 mmol), Na_2_absa (0.1 mmol) and bipy (0.1 mmol). For compound **3**, IR (KBr, cm^−1^): 3064, 1611, 1569, 1483, 1426, 1385, 1362, 1293, 1258, 1220, 1187, 1073, 832, 805, 783, 722, 642, 587, 457. Calculated for C_24_H_18_N_4_O_7_Zn (%): C, 53.35; H, 3.33; N, 10.37. Found (%): C, 53.41; H, 3.40; N, 10.35. For compound **4**, IR (KBr, cm^−1^): 3276, 1591, 1560, 1444, 1352, 1265, 1211, 1063, 807, 673, 630, 587, 465. Calculated for C_24_H_18_N_4_O_7_Co (%): C, 52.23; H, 3.63; N, 10.16. Found (%): C, 52.20; H, 3.65; N, 10.25. For compound **5**, IR(KBr, cm^−1^): 3066, 1569, 1477, 1436, 1379, 1328, 1254, 1185, 1071, 807, 681, 638, 587, 477. Calculated for C_24_H_18_N_4_O_7_Cu (%): C, 53.54; H, 3.35; N, 10.41. Found (%): C, 53.57; H, 3.46; N, 10.65.

### 2.5. Crystallographic data and structure refinements

For compounds **1**, **6** and **7**, the measurement device used was a Rigaku Saturn 70 CCD; scan mode: ω; data collection, cell refinement and data reduction were carried out using *CrysAlisPro* (Agilent, 2012 ▸). For compounds **2**–**4**, the measurement device was a Bruker APEX-II CCD; scan mode: φ–ω; data collection was carried out using *APEX2* (Bruker, 2019 ▸); cell refinement and data reduction were carried out using *SAINT* (Bruker, 2019 ▸). For compound **5**, the measurement device was a Bruker D8 VENTURE TXS PHOTON 100; scan mode: φ–ω; data collection was carried out using *APEX2* (Bruker, 2019 ▸); cell refinement and data reduction were carried out using *SAINT* (Bruker, 2019 ▸). For compounds **1**–**7**, the program used to solve the structure by the dual-space method was *SHELXT* (Sheldrick, 2015*a* ▸) and the program used to refine the structure was *SHELXL* (Sheldrick, 2015*b* ▸).

Crystallographic data were deposited in the Cambridge Crystallographic Data Centre (CCDC 2210140–2210146). The data can be obtained free of charge from https://www.ccdc.cam.ac.uk/conts/retrieving.html or on request by contacting deposit@ccdc.cam.ac.uk.

## 3. Results and discussion

### 3.1. Structural characterization

**Figure 1:**
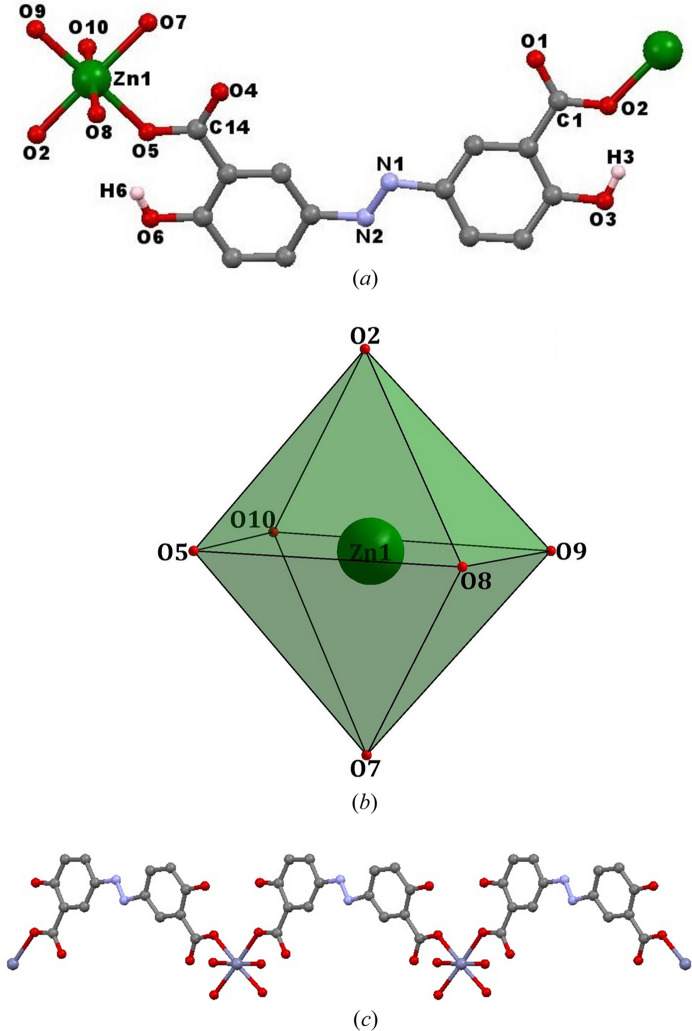
(*a*) Molecular structure of compound **1** showing the atomic numbering schemes. All hydrogen atoms, with the exception of H3 and H6, have been omitted for clarity. (*b*) Coordination configuration of Zn1 ion. (*c*) One-dimensional chain structure.

Single-crystal X-ray analysis reveals that compounds **1** and **2** have similar structures, although their space groups and unit cells are different. Therefore, only the structure of compound **1** is described in detail. Compound **1** crystallizes in the space group *P*2_1_/*c* with the monoclinic system (Table S1 of the supporting information). The asymmetric unit comprises one Zn atom, one absa^2−^ ligand and four coordinated water molecules [Fig. 1 ▸(*a*)]. Each Zn atom is in a distorted octahedron [Fig. 1 ▸(*b*)]. The absa^2−^ ligands bridge with the Zn ions to generate a chain structure through the carboxyl­ate groups in a monodentate coordination fashion [Fig. 1 ▸(*c*)]. Note however that both phenol groups of the absa^2−^ ligand are not coordinated to Zn ions which, in the protonated state, balance the charges in compound **1**.

**Figure 2:**
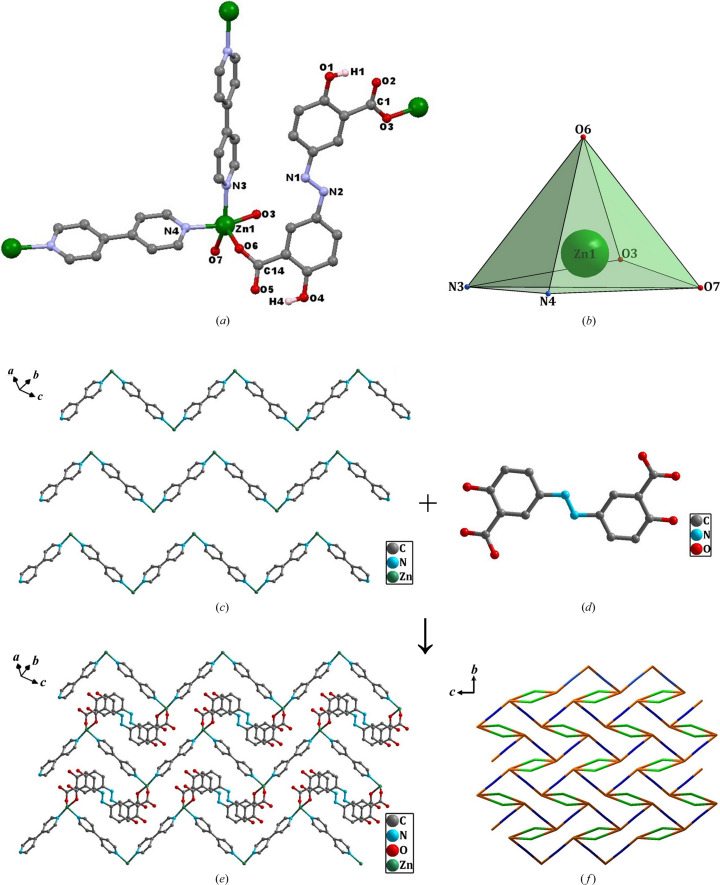
(*a*) Molecular structure of compound **3** showing the atomic numbering schemes. All hydrogen atoms, with the exception of H1 and H4, have been omitted for clarity. (*b*) Coordination configuration of the Zn1 ion. (*c*) Wave-like one-dimensional chain structure based on bipy molecules and Zn ions. (*d*) Bridging absa^2−^ ligand. (*e*) Two-dimensional network. (*f*) Four-connected framework.

The structures of compounds **3**–**5** are similar, hence only compound **3**, as an example, is described in detail. Single-crystal X-ray analysis revealed that compound **3** crystallizes in the triclinic space group *P* 1 (Table S3). Its asymmetric unit consists of one crystallographically independent Zn atom, one absa^2−^ ligand, one bipy molecule and one coordinated water molecule [Fig. 2 ▸(*a*)]. Each Zn atom adopts a distorted square-pyramidal coordination geometry [Fig. 2 ▸(*b*)]. The bipy molecule binds to the Zn ions, acting as a typical bridging ligand (Table S4). Each absa^2−^ ligand binds to Zn ions in bridging mode through both monodentate carboxyl­ate groups, leaving both protonated phenol groups uncoordinated. Each bipy molecule bridges with Zn ions to form a wave-like one dimensional chain structure [Fig. 2 ▸(*c*)], and the absa^2−^ ligands link these one-dimensional chains to generate a two dimensional network through the coordination of carboxyl­ate groups with Zn ions [Figs. 2 ▸(*d*) and 2 ▸(*e*)].

To gain better insight into the framework structure, a topological analysis was carried out. Zn atoms bind to two absa^2−^ and two bipy ligands, and thus can be simplified as four-connected nodes, with the absa^2−^ and bipy ligands acting as connecting rods. The overall topology can be described as a four-connected framework [Fig. 2 ▸(*f*)]. From a topological point of view, it exhibits a two-dimensional layered net with the Schläfli symbol (4^3^·6^3^).

### 3.2. Comparison between compounds **1**–**5**

Compounds **1**–**5** were synthesized by the application of a magnetic field. Compounds **1** and **2** have similar one-dimensional structures, which crystallize in the space group *P*2_1_/*c* with the monoclinic system, and space group * P*4_3_2_1_2 with the tetragonal system, respectively. Compounds **3**–**5** have similar two-dimensional frameworks, which crystallize in the triclinic space group *P* 1, and monoclinic space group *P*2_1_/*n*. Note that their architectures are similar, but there is a slight difference in the lattice packing. Magnetic fields can cause the interesting phenomena observed in this work. Therefore, it is necessary to compare the magnetic field effects in the packings and framework geometries. As reported in the literature, on the one hand, the presence of a magnetic field has a significant influence on the intermolecular interactions of coordination polymers (Zubir *et al.*, 2018 ▸). The molecules with π-conjugated systems lie parallel to the substrate for the sample grown under a magnetic field and slightly tilted for the sample without a magnetic field (Kolotovska *et al.*, 2006 ▸); on the other hand, there are noticeable changes in the morphology of irregular agglomerates at zero field to regular crystals with smooth surfaces under a magnetic field (Zubir *et al.*, 2016 ▸). Therefore, in contrast to zero magnetic field, a magnetic field can bring crystal orientation and morphology changes of coordination polymers, and increase the symmetry of crystal structures (Zubir *et al.*, 2018 ▸). In addition, single-crystal X-ray analyses reveal that the average bond lengths of Zn—O and Co—O in compounds **1** and **2** are 2.091 and 2.179 Å (Table S2), and 2.012 Å (Zn—O) and 2.114 Å (Co—O) in compounds **3** and **4** (Table S4), respectively. Magnetic fields can strengthen the bonding interaction through induction interactions (Hong *et al.*, 2019 ▸). As a consequence, Zn—O and Co—O bonds in compounds **3** and **4** are stronger than those in compounds **1** and **2**. This phenomenon may be attributed to the different interactions of the magnetic field with paramagnetic centers and antimagnetic organic molecules (Hong *et al.*, 2019 ▸), which provide an effective pathway for structural design of molecules, and even desired physical–chemical properties.

### 3.3. Influence of magnetic fields

Magnetic fields have been applied in materials research fields and achieved unexpected results (Gelfgat, 1999 ▸; Lin *et al.*, 2014 ▸; Serantes *et al.*, 2018 ▸). Introduction of a magnetic field can cause changes in magnetic orientation, mass-transport and concentration. In the preparation and self-assembly behavior, it can play an important role in increasing the directionality and collision probability of moving microparticles, and hence generate new materials (Xing *et al.*, 2009 ▸, 2007 ▸). Consequently, the morphologies, structures, sizes and properties of materials can be drastically modified, and the purity and crystallinity of materials have both shown marked improvement (Xing *et al.*, 2007 ▸; Wu *et al.*, 2005 ▸). The induction of the magnetic field is suggested to be a promising method for the preparation of novel structures. Therefore, we propose the introduction of a magnetic field into the crystal synthetic approach.

As is known, water is an important polar solvent for chemical reactions, in which electrolytes can be dissolved to form anions and cations (Li *et al.*, 2008 ▸). In solution, ligand and metal cations are affected by the thermal movement of water molecules around them, and they move and collide irregularly. Therefore, the self-assembly of ligands and metal cations was induced by the intermolecular weak interactions and coordination bonds to construct the coordinaiton compounds (Lu *et al.*, 2003 ▸). However, it is emphasized that the different structural characteristics of the coordination polymers can be mainly attributed to two main factors in this work. On the one hand, magnetic effect is essential for the well aligned orientation of aromatic ligand molecules (Morii *et al.*, 2005 ▸, 2004 ▸). In addition, a constant magnetic field influences both nucleation and growth of crystals of coordination polymers in a convection-free environment (Gavira & García-Ruiz, 2009 ▸). When the linear magnetic field is applied, the planes of the π-system in aromatic ligands are theoretically expected to orientate only perpendicular to the magnetic field in divergent directions, and the movement behaviors of metal cations and ligand anions are changed. The orientation and movement behaviors under a magnetic field can make the collision probability of the components in the specific direction much higher than other directions, which in turn affects the packing and coordination modes of the ligands with the metal cations (Li *et al.*, 2017 ▸). It would be preferable to generate crystal nucleation to grow a specific structure (Gavira & García-Ruiz, 2009 ▸). With the evaporation of the solvent, crystal nucleii can gradually precipitate from solution and grow into block crystals suitable for single-crystal X-ray diffraction.

**Figure 3:**
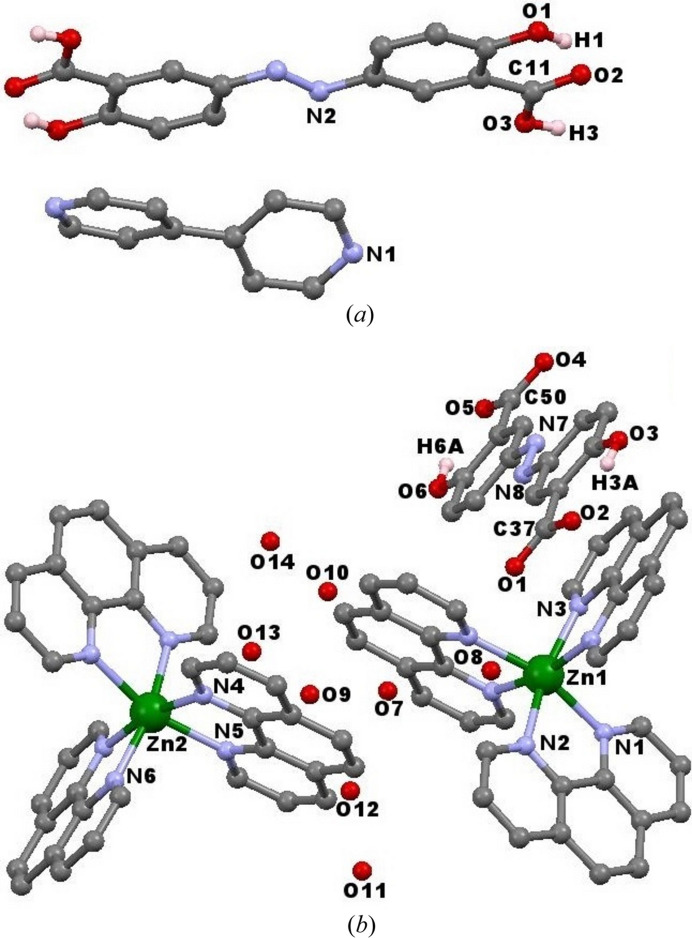
Molecular structures of (*a*) compound **6** and (*b*) compound **7** (mononuclear N-donor coordination compound) showing the atomic numbering schemes.

In order to better explain the result, we conducted comparative experiments. Under zero magnetic field, (H_2_absa)·(bipy) (**6**) is separated from the solution, where the absa^2−^ ligand and bipy molecule are uncoordinated from the metal ions [Fig. 3 ▸(*a*) and Table S5]. This may be attributed to the fact that the carboxyl­ate groups in the H_2_absa ligand are protonated and bind to fewer metal centers.

With the increase of the magnetic field to 0.3 T, phen molecules, acting as chelated terminated ligands, prefer to coordinate with metal ions and occupy all coordination sites, in contrast to absa^2−^ ligands; thus a mononuclear coordination compound with N-donor ligands [Zn(phen)_3_]·(absa)·7H_2_O (**7**) was obtained [Fig. 3 ▸(*b*) and Table S5]. Under a 1 T magnetic field, the absa^2−^ ligands can resist irregular movement to a certain extent, and tend to be perpendicular to the magnetic field. Thus, they gain the opportunity to approach metal ions to form one-dimensional chains. When bipy is added under magnetic field, the absa^2−^ ligand is perpendicular to the magnetic field, which makes the ligand coordinate with metal ions in a certain direction, generating a two-dimensional net.

Very few block crystals of compounds **1**–**5** were isolated. Many attempts were made to obtain more crystals by improving the reaction conditions, but were unsuccessful. Because not enough samples were available, additional measurements were not performed, but thermogravimetric analyses and photoluminescence properties were studied. The effect of magnetic field on the types of structures are rather complicated and difficult to discuss. However, it is worth noting that the metal sources and the auxiliary ligands also play an important role. The orientation of the absa^2−^ ligand in a magnetic field will affect its coordination mode in compounds **1** and **2** relative to compounds **6** and **7**. The presence of a high magnetic field and bipy facilitates the formation of high-dimensional coordination polymers such as compounds **3**–**5**. However, much more systematic work is needed to further elucidate the magnetic field effect, which can influence and induce the formation of the resultant coordination compounds. It is also anticipated that further new types of coordination polymers can be designed by this synthetic method.

### 3.4. Thermogravimetric analyses

**Figure 4:**
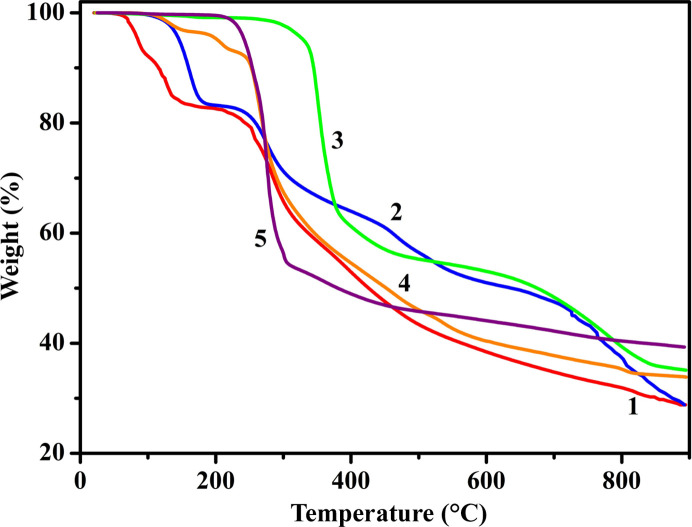
TGA curves of compounds **1**–**5**.

To investigate the thermal stability of compounds **1**–**5**, TGA was performed under a nitro­gen atmosphere at the heating rate of 10°C min^−1^ between 25 and 900°C (Fig. 4 ▸). The TGA curves of compounds **1** and **2** exhibit the first weight losses of 16.7 and 16.6% in the temperature ranges 25–156°C and 25–189°C, which correspond to the release of coordinated water molecules (calculated 16.5 and 16.7%, respectively). The second weight losses in the temperature ranges 156–900°C and 189–900°C correspond to the pyrolysis of the absa^2−^ ligands. Up to 900°C, the thermogravimetric curves still show downward trends. The residues may be the metal oxide. For compounds **3**–**5**, the weight losses in the temperature ranges 25–315°C, 25–220°C and 25–237°C amount to 3.4, 6.7 and 3.4%, which can be attributed to the removal of the coordinated water molecules (calculated 3.3, 6.5 and 3.3%, respectively). The weight losses of 29.0, 28.5 and 29.5% in the temperature ranges 315–371°C, 220–313°C and 237–280°C correspond to the loss of the bipy molecules (calculated 28.9, 28.3 and 29.0%, respectively). The weight losses in the temperature ranges 371–900°C, 313–900°C and 280–900°C correspond to the decomposing of the absa^2−^ ligands. Up to 900°C, the thermogravimetric curves still show downward trends. The residues may be the metal oxide.

### 3.5. Photoluminescence properties

**Figure 5:**
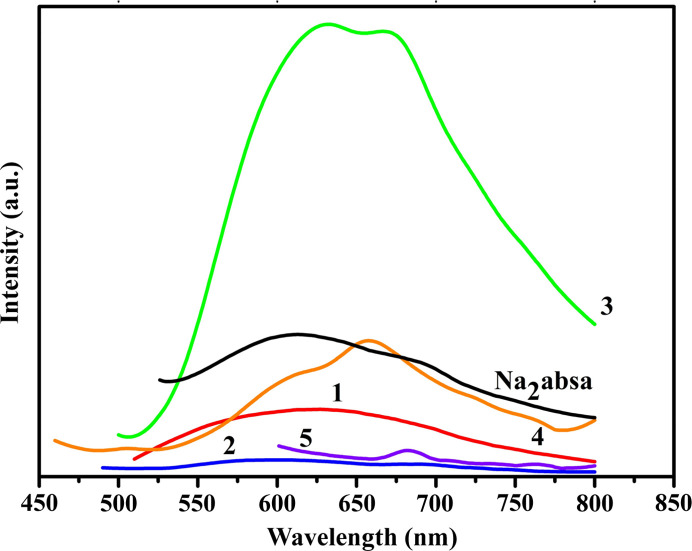
Solid-state emission spectra of Na_2_absa and compounds **1**–**5** at room temperature.

Luminescent coordination polymers are currently of great interest because of their applications in photochemistry, chemical sensors and luminescent displays (Allendorf *et al.*, 2009 ▸). To establish the relationship between the crystal structures and their fluorescence properties, the solid fluorescence spectra of compounds **1**–**5** as well as the free Na_2_absa ligand for comparison were measured at room temperature (Fig. 5 ▸). Compounds **1**–**5** exhibit photoluminescence emissions at 626, 600, 632, 658 and 682 nm, respectively, which are similar to those of the free Na_2_absa ligand with an emission maximum at 612 nm. The observed emissions in compounds **1**–**5** are probably caused by π–π* intra-ligand transitions of the Na_2_absa ligand (Cheng *et al.*, 2012 ▸). The observed red and blue shifts of the emissions maximum between compounds **1**–**5** and the Na_2_absa ligand were mainly attributed to the coordination of ligands with metal ions (Zhang *et al.*, 2014 ▸). However, it is found that the emission intensity of compound **3** is much higher than those of compounds **1**, **2**, **4** and **5**. This indicates that the zinc compound **3** shows better photoluminescence properties than the others. Whereas the emission intensities of compounds **1**, **2**, **4** and **5** are much lower than those of the free Na_2_absa ligand, which indicates that the emission of the Na_2_absa ligand was quenched to some extent.

The magnetic field assisted self-assembly based on transition metal ions and the absa^2−^ ligand in the presence and absence of nitro­gen donor molecules leads to the formation of coordination polymers with different characteristics. They feature one-dimensional and two-dimensional structures with different space groups, respectively. The progressive structural characteristics result from the distinct orientation of ligands and the movement behavior of components, leading to packing in certain directions as a consequence of the effects of magnetic fields. In addition, compounds **1**–**5** exhibit broad fluorescent emission bands at 626, 600, 632, 658 and 682 nm, respectively.

## Supplementary Material

Crystal structure: contains datablock(s) compound_1, compound_2, compound_3, compound_4, compound_5, compound_6, compound_7. DOI: 10.1107/S2052252523007650/ed5029sup1.cif

Supporting tables. DOI: 10.1107/S2052252523007650/ed5029sup2.pdf

CCDC references: 2210140, 2210141, 2210142, 2210143, 2210144, 2210145, 2210146
