# Supplementary material for: A new route for the syntheses of coordination polymers using magnetic influence: syntheses, crystal structures and fluorescence properties
Source: IUCrJ. 2023 Sep 18;10(Pt 6):671–7. doi: 10.1107/S2052252523007650 (PMC10619453; doi:10.1107/S2052252523007650)
Supplement: Supplementary file 2 [file m-10-00671-sup2.pdf]

IUCrJ

Volume 10 (2023)

Supporting information for article:

A new route for the syntheses of coordination polymers and magnetic influence: syntheses, crystal structures and fluorescence properties

Lei Guan, Ying Wang, Hongzhe Jin and Pengpeng Yin

Table S1 Crystallographic data and structure refinement for compounds **1-2**

	**1**	**2**
Chemical formula	$C_{14}H_{16}N_2O_{10}Zn$	$C_{14}H_{16}N_2O_{10}Co$
M_r	437.66	431.22
Crystal system, space group	Monoclinic, $P2_1/c$	Tetragonal, $P4_32_12$
Temperature (K)	113	193
a, *b*, *c* (Å)	9.4322(4), 11.3669(4), 16.0822(7)	10.2539(5), 10.2539(5),30.4058 (15)
α, β, γ(°)	106.000(4)	90, 90, 90
V (Å^3)	1657.46(12)	3196.9(3)
Z	4	8
Radiation type	Mo-K_α	Mo-K_α
μ (mm^{-1})	1.54	6.27
Crystal size (mm)	0.16 × 0.13 × 0.1	0.15×0.12×0.13
No. of measured, independent and observed [$I > 2\sigma(I)$] reflections	20581, 5723, 4485	30178, 2881, 2787
R_{int}	0.059	0.04
$(\sin \theta/\lambda)_{max}$ (Å^{-1})	0.764	0.603
$R[F^2 > 2\sigma(F^2)]$, $wR(F^2)$, S	0.052, 0.126, 1.10	0.030, 0.096, 0.86
No. of reflections	5723	2881
No. of parameters	249	255
No. of restraints	0	0
$\Delta\rho_{max}$, $\Delta\rho_{min}$ (e·Å^{-3})	0.75, −0.72	0.31, −0.54

Table S2 Selected bond lengths (Å) and bond angles (deg) for compounds **1-2**

		**1**	
Zn1—O2^{i}	2.100(2)	Zn1—O8	2.082(2)
Zn1—O5	2.0991(19)	Zn1—O9	2.077(2)
Zn1—O7	2.124(2)	Zn1—O10	2.064(2)
O2^{i}—Zn1—O7	176.64(9)	O9—Zn1—O7	87.92(8)
O5—Zn1—O2^{i}	85.87(8)	O9—Zn1—O8	89.17(8)
O5—Zn1—O7	92.68(8)	O10—Zn1—O2^{i}	88.30(9)
O8—Zn1—O2^{i}	90.46(9)	O10—Zn1—O5	90.00(8)
O8—Zn1—O5	87.87(8)	O10—Zn1—O7	88.66(9)
O8—Zn1—O7	92.52(9)	O10—Zn1—O8	177.61(8)
O9—Zn1—O2^{i}	93.68(8)	O10—Zn1—O9	92.96(8)
O9—Zn1—O5	177.00(8)		
Symmetry code: (i) x+1, y+1, z.			
		**2**	
Co01—O9	2.141(2)	Co01—O2	2.168(2)
Co01—O6^{i}	2.155(2)	Co01—O8	2.219(2)
Co01—O7	2.156(3)	Co01—O10	2.234(3)
O9—Co01—O6^{i}	86.63(10)	O7—Co01—O8	88.14(10)
O9—Co01—O7	92.40(10)	O2—Co01—O8	99.47(10)
O6^{i}—Co01—O7	106.08(11)	O9—Co01—O10	92.19(10)
O9—Co01—O2	173.41(10)	O6^{i}—Co01—O10	85.82(10)
O6^{i}—Co01—O2	86.98(9)	O7—Co01—O10	167.47(11)
O7—Co01—O2	87.83(10)	O2—Co01—O10	88.93(10)
O9—Co01—O8	87.12(10)	O8—Co01—O10	80.46(9)
O6^{i}—Co01—O8	164.68(10)		
Symmetry code: (i) x−1, y−1, z.			

Table S3 Crystallographic data and structure refinement for compounds **3-5**

	**3**	**4**	**5**
Chemical formula	$C_{24}H_{18}N_4O_7Zn$	$C_{24}H_{20}N_4O_8Co$	$C_{24}H_{18}N_4O_7Cu$
M_r	539.79	551.37	537.96
Crystal system, space group	Triclinic, *P*-1	Triclinic, P-1	Monoclinic, $P2_1/n$
Temperature (K)	296	296	193
a, *b*, *c* (Å)	8.5332(2), 9.6081(2), 14.1363(3)	7.7901(4), 10.0895 (7), 15.5233(8)	7.2074(4), 16.8579 (7), 18.3599(7)
α, β, γ(°)	95.3200(10), 97.1030 (10), 104.4730(10)	94.751(2), 101.411 (2), 107.809(2)	90, 100.371(2), 90
V (Å^3)	1104.36(4)	1125.17(11)	2194.31(17)
Z	2	2	4
Radiation type	Mo-K_α	Mo-K_α	Mo-K_α
μ (mm^{-1})	1.169	0.82	1.05
Crystal size (mm)	0.1 × 0.13 × 0.15	0.12 × 0.18 × 0.16	0.12 × 0.1 × 0.1
No. of measured, independent and observed [$I > 2\sigma(I)$] reflections	10598, 5032, 4595	10738, 5141, 4509	5029, 5029, 3908
R_{int}	0.0227	0.033	0.07
$(\sin \theta/\lambda)_{max}$ (Å^{-1})	0.0311	0.651	0.649
$R[F^2 > 2\sigma(F^2)]$, $wR(F^2)$, S	0.035, 0.109, 0.115	0.035, 0.121, 0.87	0.047, 0.104, 1.07
No. of reflections	10598	5141	5029
No. of parameters	329	336	330
No. of restraints	0	0	1
$\Delta\rho_{max}$, $\Delta\rho_{min}$ (e·Å^{-3})	0.373, −0.345	0.54, −0.58	0.46, −0.40

Table S4 Selected bond lengths (Å) and bond angles (deg) for compounds **3-5**

		**3**	
Zn—N3	2.1747(16)	Zn1—O6	1.9699(14)
Zn1—N4	2.0816(16)	Zn1—O7	2.0658(16)
Zn1^{i}—O3	1.9998(13)		
O6—Zn1—O3^{i}	104.64(6)	O7—Zn1—N4	86.46(6)
O6—Zn1—O7	103.04(7)	O6—Zn1—N3	108.20(6)
O3^{i}—Zn1—O7	88.49(6)	O3^{i}—Zn1—N3	84.07(5)
O6—Zn1—N4	96.80(6)	O7—Zn1—N3	148.76(7)
O3^{i}—Zn1—N4	158.56(7)	N4—Zn1—N3	89.52(6)
Symmetry code: (i) $-x+1$, $-y+2$, $-z+1$.			
		**4**	
Co1—O1	2.0655(14)	Co1—N2	2.1173(17)
Co1—O6^{i}	2.0783(13)	Co1—N1	2.1418(15)
Co1—O7	2.1016(14)	Co1—O8	2.2142(13)
O1—Co1—O6^{i}	92.02(6)	O7—Co1—N1	93.27(6)
O1—Co1—O7	88.63(6)	N2—Co1—N1	97.44(6)
O6^{i}—Co1—O7	175.67(5)	O1—Co1—O8	89.16(5)
O1—Co1—N2	176.31(5)	O6^{i}—Co1—O8	87.31(5)
O6^{i}—Co1—N2	90.26(6)	O7—Co1—O8	88.41(5)
O7—Co1—N2	88.88(6)	N2—Co1—O8	88.05(6)
O1—Co1—N1	85.42(6)	N1—Co1—O8	174.28(6)
O6^{i}—Co1—N1	91.05(6)		
Symmetry code: (i) $-x$, $-y$, $-z+1$.			
		**5**	
Cu1—O3^{i}	2.257(2)	Cu1—N1	2.018(2)
Cu1—O6	1.9496(19)	Cu1—N2^{ii}	2.024(2)
Cu1—O7	1.935(2)		
O6—Cu1—O3^{i}	97.40(9)	O7—Cu1—N1	88.86(9)
O6—Cu1—N1	168.57(11)	O7—Cu1—N2^{ii}	169.39(12)
O6—Cu1—N2^{ii}	86.49(8)	N1—Cu1—O3^{i}	94.00(9)
O7—Cu1—O3^{i}	99.65(11)	N1—Cu1—N2^{ii}	94.30(9)
O7—Cu1—O6	88.43(8)	N2^{ii}—Cu1—O3^{i}	90.24(9)
Symmetry codes: (i) $-x+1$, $-y+1$, $-z+1$; (ii) $-x+1/2$, $y+1/2$, $-z+3/2$.			

Table S5 Crystallographic data and structure refinement for compounds **6-7**

	**6**	**7**
Chemical formula	$C_{24}H_{18}N_4O_6$	$C_{50}H_{48}N_8O_{14}Zn$
M_r	458.42	1050.33
Crystal system, space group	Monoclinic, *C*2/*c*	Monoclinic, *P*2/*c*
Temperature (K)	173	113
a, *b*, *c* (Å)	12.020(2), 11.953(2), 14.174(3)	14.1895(10), 14.8309(9), 22.4020(15)
α, β, γ(°)	90, 103.163(7), 90	90, 92.676(6), 90
V (Å^3)	1983.1(7)	4709.2(5)
Z	4	4
Radiation type	Mo-K_α	Mo-K_α
μ (mm^{-1})	0.11	0.6
Crystal size (mm)	0.13 × 0.12 × 0.1	0.12 × 0.1 × 0.07
No. of measured, independent and observed [$I > 2\sigma(I)$] reflections	8494, 2265, 2018	41394, 9636, 5147
R_{int}	0.044	0.141
$(\sin \theta/\lambda)_{max}$ (Å^{-1})	0.65	0.625
$R[F^2 > 2\sigma(F^2)]$, $wR(F^2)$, S	0.046, 0.129, 1.07	0.065, 0.131, 1.00
No. of reflections	2265	9636
No. of parameters	156	692
No. of restraints	0	11
$\Delta\rho_{max}$, $\Delta\rho_{min}$ (e·Å^{-3})	0.46, −0.30	0.54, −0.53
